# Low frequency of mismatch repair deficiency in gallbladder cancer

**DOI:** 10.1186/s13000-019-0813-5

**Published:** 2019-05-08

**Authors:** Benjamin Goeppert, Stephanie Roessler, Marcus Renner, Moritz Loeffler, Stephan Singer, Melina Rausch, Thomas Albrecht, Arianeb Mehrabi, Monika Nadja Vogel, Anita Pathil, Elena Czink, Bruno Köhler, Christoph Springfeld, Christian Rupp, Karl Heinz Weiss, Peter Schirmacher, Magnus von Knebel Doeberitz, Matthias Kloor

**Affiliations:** 1 Institute of Pathology, University Hospital Heidelberg, Im Neuenheimer Feld 224, Heidelberg, Germany; 2 Liver Cancer Center Heidelberg (LCCH), Heidelberg, Germany; 3 Department of General Visceral and Transplantation Surgery, University Hospital Heidelberg, Im Neuenheimer Feld 110, Heidelberg, Germany; 4 Diagnostic and Interventional Radiology, Thoraxklinik at University Hospital of Heidelberg, Heidelberg, Germany; 5 Department of Internal Medicine IV, Gastroenterology and Hepatology, University Hospital Heidelberg, Im Neuenheimer Feld 410, Heidelberg, Germany; 6 Department of Medical Oncology, National Center for Tumor Diseases, University Hospital Heidelberg, Heidelberg, Germany; 7 Department of Applied Tumor Biology, Institute of Pathology, University of Heidelberg, Heidelberg, Germany

**Keywords:** Biliary tract cancer, Gallbladder cancer, DNA mismatch repair deficiency, Microsatellite instability

## Abstract

**Background:**

DNA mismatch repair (MMR) deficiency is a major pathway of genomic instability in cancer. It leads to the accumulation of numerous mutations predominantly at microsatellite sequences, a phenotype known as microsatellite instability (MSI). MSI tumors have a distinct clinical behavior and commonly respond well to immune checkpoint blockade, irrespective of their origin. Data about the prevalence of MSI among gallbladder cancer (GBC) have been conflicting. We here analyzed a well-characterized cohort of 69 Western-world GBCs.

**Methods:**

We analyzed the mononucleotide MSI marker panel consisting of BAT25, BAT26, and CAT25 to determine the prevalence of MMR deficiency-induced MSI.

**Results:**

MSI was detected in 1/69 (1.4%) of analyzed GBCs. The detected MSI GBC had a classical histomorphology, i.e. of acinar/tubular/glandular pancreatobiliary phenotype, and showed nuclear expression of all four MMR proteins MLH1, MSH2, MSH6, and PMS2. The MSI GBC patient showed a prolonged overall survival, despite having a high tumor stage at diagnosis. The patient had no known background or family history indicative of Lynch syndrome.

**Conclusions:**

Even though the overall number of MSI tumors is low in GBC, the potentially therapeutic benefit of checkpoint blockade in the respective patients may justify MSI analysis of GBC.

**Electronic supplementary material:**

The online version of this article (10.1186/s13000-019-0813-5) contains supplementary material, which is available to authorized users.

## Introduction

The incidence of DNA mismatch repair (MMR)-deficient, microsatellite-unstable (MSI) tumors is of particular clinical relevance since it has been shown that MMR-deficient tumors are more responsive to immune checkpoint blockade, particularly using antibodies directed against the immune checkpoint PD-1 or PD-L1 [1], than MMR-proficient tumors. Histomorphologically, gallbladder cancer (GBC) is a diverse group of tumors, most often displaying phenotypes of an adenocarcinoma. Clinically, GBC is often treated together with cholangiocarcinomas (CCAs) as biliary tract cancers and thus as one disease, although genetic heterogeneity as well as differences in clinical behavior is nowadays widely acknowledged [2]. Most patients with GBC present with unresectable or metastatic disease. Despite systemic chemotherapy, prognosis remains poor and to date there are no established molecular targeted therapies tailored to GBC [3]. Molecular events occurring during the development of biliary tract cancers (BTC) are heterogeneous and likely follow a multistep process encompassing alterations of several oncogenes and tumor suppressor genes, such as *KRAS, TP53, EGFR,* and *ERBB2/3*. The genomic spectra of BTC have been previously nicely depicted [4], and we have recently demonstrated the presence of MSI in a small proportion (4/308; 1.3%) of CCAs [5].

So far, there are no systematic data available informing about the prevalence of DNA mismatch repair deficiency and MSI in GBC. Previous studies on MSI in biliary tract cancers have reported MMR deficiency and MSI in up to 30% of cases, particularly in liver-fluke-associated CCA specimens from endemic regions in Thailand [6]. For GBC, reported proportions of MSI tumors also varied greatly, because of a high diversity of marker panels used for determining MSI.

In the present study, we aimed to determine frequency and clinicopathological characteristics of MSI tumors in a well-characterized German cohort of GBC.

## Methods

### Clinicopathological characteristics of gallbladder carcinoma patients

Resection specimens from 69 patients (mean age: 69.8 years, median age: 71.4 years) who underwent hepatobiliary surgery at the University Hospital Heidelberg between 1995 and 2010 were included in this study. The GBC cohort consisted only of adenocarcinomas, including all histologic variants. None of the patients received radio- and/or chemotherapy prior to surgery. Tumors were restaged according to the 8th TNM Classification of Malignant Tumors and classified according to the current World Health Organization (WHO) tumor classification system (WHO Classification of Tumours of the Digestive System, 4th Ed., 2010) by two experienced pathologists, i.e. consultant pathologists of our institute with a specialization in hepatobiliary pathology (BG and SS). A summary of clinicopathological data is given in Table 1. The use of the tissues for this study was approved by the institutional ethics committee (206/05).

**Table 1:** *Clinicopathological data of the gallbladder carcinoma cohort with complete clinicopathological data and correlation to the MSI status (n = 69)*

All GBC patients		Number (percent	Number (percent)	Number (percent)
		total	non-MSI	MSI
		69 (100.0)	68 (98.6)	1 (1.4)
*Age* ^*a*^				
*< 69.8 yrs*		28 (40.6)	27 (39.1)	*1 (1.4)*
*> 69.8 yrs*		41 (59.4)	41 (59.4)	0 (0.0)
Sex				
*m*		21 (30.4)	21 (30.4)	0 (0.0)
*w*		48 (69.6)	47 (68.1)	1 (1.4)
pT				
*T1*		5 (7.2)	5 (7.2)	0 (0.0)
*T2*		30 (43.5)	29 (42.0)	1 (1.4)
*T3*		26 (37.7)	26 (37.7)	0 (0.0)
*T4*		8 (11.6)	8 (11.6)	0 (0.0)
pN				
*N0*		13 (18.8)	13 (18.8)	0 (0.0)
*N1*		19 (27.5)	18 (26.1)	1 (1.4)
*Nx*		37 (53.6)	37 (53.6)	0 (0.0)
M				
*M0*		56 (81.2)	55 (79.7)	1 (1.4)
*M1*		13 (18.8)	13 (18.8)	0 (0.0)
UICC^b^				
*UICC 2*		5 (7.2)	5 (7.2)	0 (0.0)
*UICC 3*		18 (26.1)	17 (24.6)	1 (1.4)
*UICC 4*		18 (26.1)	18 (26.1)	0 (0.0)
*NA*		28 (40.6)	28 (40.6)	0 (0.0)
G				
*G1*		4 (5.8)	4 (5.8)	0 (0.0)
*G2*		41 (59.4)	40 (58.0)	1 (1.4)
*G3*		24 (34.8)	24 (34.8)	0 (0.0)
L				
*L0*		42 (60.9)	42 (60.9)	0 (0.0)
*L1*		27 (39.1)	26 (37.7)	1 (1.4)
V				
*V0*		53 (76.8)	52 (75.4)	1 (1.4)
*V1*		16 (23.2)	16 (23.2)	0 (0.0)
Pn				
*Pn0*		50 (72.5)	50 (72.5)	0 (0.0)
*Pn1*		19 (27.5)	18 (26.1)	1 (1.4)
Histology				
*ductal*		48 (69.6)	47 (68.1)	1 (1.4)
*papillary*		6 (8.7)	6 (8.7)	0 (0.0)
*mucinous*		8 (11.6)	8 (11.6)	0 (0.0)
*intestinal*		1 (1.4)	1 (1.4)	0 (0.0)
*other*		6 (8.7)	6 (8.7)	0 (0.0)

^a^Median patients’ age was 71.4 years and mean age was 69.8 years; ^b^Cases with pNx had no lymph nodes resected, therefore, UICC status could not be assessed

*pT* Pathology-based tumor stage, *pN* Pathology-based nodal status, *M* Distant metastasis status, *UICC* Tumor stage, *G* Tumor grade, *L* Lymph vessel invasion, *V* Blood vessel invasion, *Pn* Perineural invasion. The 6 tumors listed in the histology section as „other “are 3 adenosquamous carcinomas, 2 signet ring carcinomas, and 1 clear-cell adenocarcinoma

### Tissue microarray construction

From all 69 GBC FFPE tissue blocks, 3 μm sections were cut and stained with H&E. Representative areas were marked by experienced pathologists (BG and SS). For each case, tumor tissue cores (1.0 mm diameter) from the selected representative tumor areas were punched out of the sample tissue blocks and embedded into a new paraffin array block using a tissue microarrayer (Beecher Instruments, Woodland, CA, USA).

### Immunohistochemical staining of MMR proteins

Immunohistochemical analyses were performed on 3 μm thick sections in all tumors classified as MSI by PCR-based methods. Briefly, the slides were pretreated by boiling for 10 min with a microwave in target retrieval buffer (pH 9, Dako, Hamburg, Germany) before application of monoclonal antibodies specific for MSH2 (clone FE11, dilution 1:100, Calbiochem, Darmstadt, Germany), MLH1 (clone G168–15, 1:100, BD Biosciences, San Diego, CA, USA), MSH6 (clone 44; 1:200; BD Biosciences, San Diego, CA, USA), and PMS2 (clone EPR3947, ready to use; Cell Marque, Sigma Aldrich, St. Louis, Missouri, USA). An immunoperoxidase method was used to visualize bound antibodies with DAB (3-amino-9-ethylcarbazole, Dako) as chromogen.

### Molecular microsatellite instability analysis

DNA was extracted using the DNeasy Blood and Tissue kit (Qiagen, Hilden, Germany) and eluted in pure water. PCR with fluorescence-labelled oligonucleotides was performed to amplify the mononucleotide markers BAT25, BAT26, and CAT25 from tumor tissue DNA as described previously [7]. From the tumor classified as MSI, in addition BAT40 was amplified, using the oligonucleotide primers 5′-ATTAACTTCCTACACCACAAC-3′ and 5′-gTAgAgCAAgACCACCTTg-3′. Amplified fragments were visualized on an ABI3130xl genetic analyzer (Applied Biosystems, Darmstadt, Germany) to detect potential length alterations of the microsatellites. Fragment sizes differing from the normal range of allelic size variation known for the amplicons encompassing BAT25 (108–110 bp), BAT26 (116–118 bp), and CAT25 (146–148 bp) were regarded as MSI [7]. Tumors showing such amplicons in two or more markers were classified as MSI.

### Statistical analyses including correlation analyses with other immunohistochemical variables

Statistical analyses were performed with the statistical computing environment R version 3.0.1. Correlation analyses of MSI status with clinicopathological and other immunohistochemical variables were assessed with Fisher’s exact test. Due to the low number of detected MSI cases, a statistical correlation analysis was not feasible for all given parameters. Univariate survival analysis was performed for overall survival by generation of Kaplan-Meier curves using GraphPad Prism 6. Significance of differences between the groups was assessed using the Log-rank-test. *P* values < 0.05 were considered significant. Data concerning expression of PD-L1, MHC I, quantity and quality of tumor infiltrating immune cells, as well as proliferation index (Ki-67) were available partly from previously published studies [8, 9].

## Results

### Frequency of microsatellite instability in gallbladder carcinoma

**Fig. 1:**
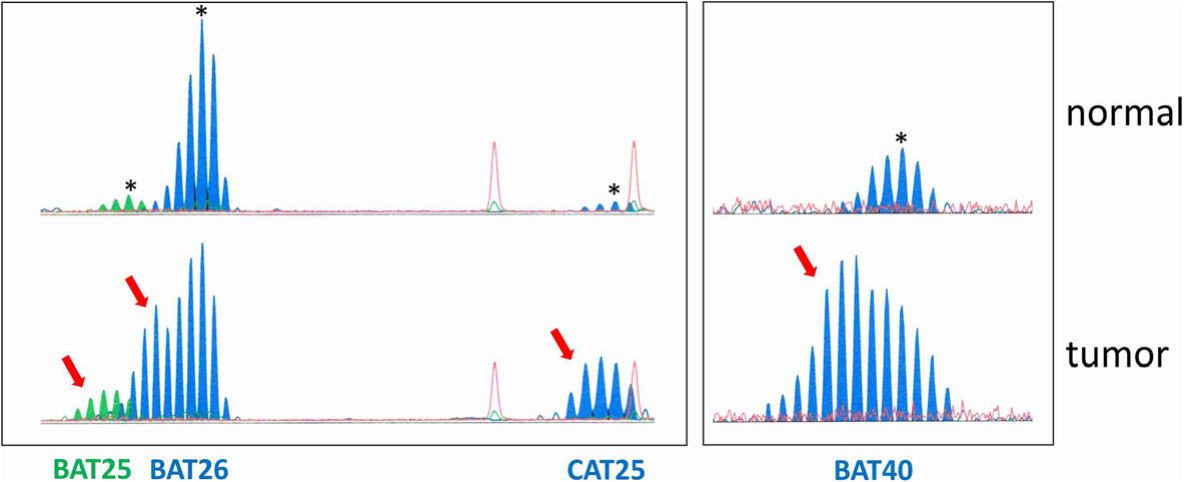
*Results of microsatellite instability analysis.* Peak patterns of the MSI tumor analyzed by the GeneMapper Software (version 5, Applied Biosystems) show aberrant peak profiles indicative of MSI in all 4 tested mononucleotide markers. Highest peaks in MSI profiles obtained from normal tissue are marked by asterisks (upper panel). Aberrant peaks detected in tumor tissue are marked by red arrows (lower panel)

**Fig. 2:**
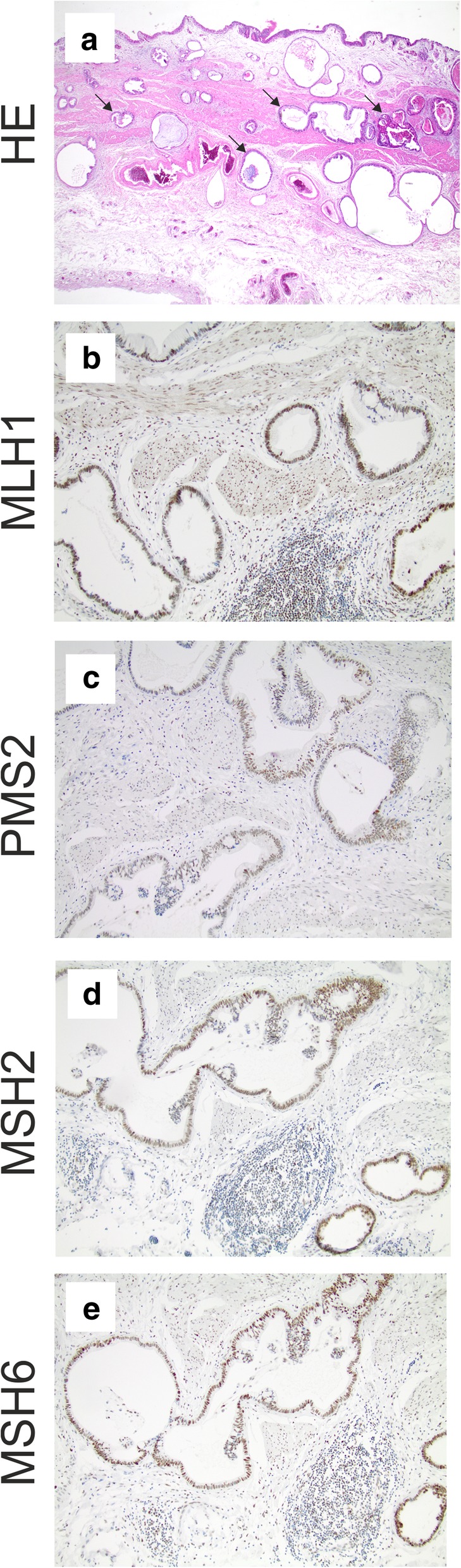
*Histology and immunohistochemistry of the detected MSI gallbladder carcinoma.* **a**: Hematoxylin & Eosin (HE) staining of a full-slide section of the detected MSI GBC shows a typical ductal morphology of pancreatobiliary subtype (representative atypical neoplastic ducts are indicated by arrows, original magnification: 20x). **b**-**e**: Immunohistochemistry of MMR proteins showed retained nuclear signals in the neoplastic epithelia (MLH1, PMS2, MSH2, and MSH6; original magnification: 100x)

All cases were tested for MSI status using a highly sensitive mononucleotide marker panel (BAT25, BAT26, CAT25), which specifically detects DNA mismatch repair deficiency-induced alterations at long mononucleotide repeats [7, 10]. One (1.4%) out of 69 analyzed GBCs showed MSI at all three markers, whereas no aberrant peaks were detected in the remaining 68 tumor specimens. MSI status was confirmed by repeating the analysis from independently isolated tumor DNA and corresponding non-tumor DNA from the same paraffin block, and by testing BAT40 as an additional marker (Fig. 1). Immunohistochemistry for DNA mismatch repair proteins MLH1, PMS2, MSH2, and MSH6 revealed no loss of nuclear immunoreactivity for all tested repair proteins (Fig. 2).

### Clinicopathological data of MSI gallbladder carcinoma and patient survival

The detected MSI GBC had a typical histomorphology, i.e. showing the typical acinar/tubular/glandular (NOS) pancreatobiliary phenotype (Fig. 2 and Additional file 1: Figure S1). Furthermore, the MSI patient was female, belonged to the younger patient group (age at diagnosis: 68 years, not significant different to mean age of the entire GBC cohort: 69.8 years), showed a positive lymph node status (pN1) and a high UICC stage (UICC stage 3; Table 1). Nevertheless, the MSI GBC patient showed an overall survival of 4.5 years after surgery, compared to a median OS in patients with non-MSI tumors of 1.6 years.

### Immune phenotype of microsatellite-unstable gallbladder carcinoma

MSI tumors commonly present with a high density of local immune cell infiltration and expression of immune checkpoint molecules. We analyzed the quantity and quality of tumor infiltrating immune cells, MHC class I, PD-L1, and HER2-status in the same GBC cohort [8, 9]. In the MSI GBC, a high expression of MHC class I antigen on the tumor cell surface could be detected (Table 2), while numbers of tumor-infiltrating immune cells were within the range of non-MSI cases of the cohort. The MSI GBC showed no PD-L1 expression, neither on the tumor cell surface nor on tumor-associated inflammatory cells. The proliferation index (Ki-67) was low (6%). Due to only one MSI GBC detected in this cohort, statistical correlation analyses for clinicopathological parameters and immune characteristics were not feasible.

**Table 2:** *MSI* vs *non-MSI gallbladder carcinomas with regard to MHC class I immunoreactivity*

MHC score	all GBC cases	MSI neg	MSI pos
0	6	6	0
1	2	2	0
2	3	3	0
3	7	7	0
4	8	8	0
6	5	5	0
8	18	17	1
9	2	2	0
12	8	8	0
NA	10	10	0
total	69	68	1

## Discussion

In summary, our study demonstrates that MMR deficiency-induced MSI is rare in this well-characterized, non-liver-fluke associated German GBC cohort (*n* = 69). The lower frequency of MSI in our study compared to several others may be explained, as previously demonstrated for CCA, but also for GBC [10–16], by regional differences and associated pathogenetic mechanisms. In addition, in the present study we exclusively used mononucleotide repeats, which are more specific for detecting DNA mismatch repair deficiency-induced MSI than di- or tetranucleotide markers [17, 18], thereby avoiding false positive results due to incidental slippage events in MMR-proficient cells. Such events may explain the high rates of MSI in the literature, which in a recent retrospective literature analysis article was 18 out of 357 (5%) in GBC [19].

In our analysis, only one (1.4%) out of 69 tumors showed instability affecting all the sensitive mononucleotide markers BAT25, BAT26, and CAT25. As immunohistochemistry did not show loss of any of the four MMR proteins analyzed, MSI analysis was repeated twice from independent tissue blocks and TMA cores, and the MSI status was confirmed. MSI tumors presenting with retained expression of MMR proteins are rare, but have been previously reported in the literature [20, 21]. Possible reasons for such a discrepancy may be somatic missense mutations in DNA mismatch repair genes that result in the expression of a non-functional MMR protein, or, as reported recently, mutations in other MMR-related genes such as the exonuclease domain of the POLE gene [20, 21]. Further analysis such as exome or whole genome sequencing of tumor tissue would have been required to identify the molecular mechanisms leading to MSI and to inform about a possible hereditary background. Unfortunately, in our case complete somatic mutation analysis of MMR genes was not feasible due to limited amounts of available tumor DNA from FFPE tissue specimens.

The classical MSI phenotype including a special MSI histology with poor differentiation and dense lymphocyte infiltration, which is known from MSI colorectal cancers [22–24], was not fully recapitulated in the detected MSI GBC. However, in line with the improved prognosis commonly reported to be associated with tumors of the MSI phenotype, the patient’s overall survival was 4.5 years, compared to a median overall survival of 1.6 years in the MSS group of the same stage (UICC 3). Due to the limited number of patients, a statistical analysis of potential survival benefits related to MSI in GBC was not feasible. A recent study, however, did not report a prolonged survival in MSI compared to non-MSI GBC patients [10].

Although the overall frequency of MSI in GBC is low with less than 2% in the present cohort, MSI testing of GBC should be considered if the patient has a chance to benefit from immune checkpoint blockade [25]. Recent studies demonstrated that patients with MSI BTC in fact may show significant clinical responses towards treatment with anti-PD-1 or anti-PD-L1 antibodies [1, 26]. Interestingly, even BTC patients with far progressed disease status that are therapy-refractory against chemotherapeutical agents commonly used in the treatment of BTC can show significant treatment responses upon immune checkpoint blockade using pembrolizumab [27]. Although we cannot state, whether MSI in GBC is associated with an increased immune infiltration due to limited patient numbers, considering the pronounced and sustainable benefit reported for advanced MSI cancer patients after treatment with immune checkpoint inhibitors, additional studies are strongly encouraged that use PCR-based MSI testing in parallel with MMR protein IHC prospectively in larger series of GBC.

Concerning MSI testing in GBC patients, this study has several clinical implications: i) it demonstrates that the frequency of MSI is low in Western-world GBC, and ii) PCR-based MSI analysis using mononucleotide markers should be considered due to their sensitivity and specificity for the detection of MMR deficiency-induced MSI, particularly in GBC patients who might be eligible for immune checkpoint blockade.

## Conclusions

The frequency of MMR deficiency-induced MSI among Western-world gallbladder cancers is low. However, due to the significant clinical responses of MSI tumor patients towards immune checkpoint blockade, MSI analysis should be considered in this tumor type.

## Additional file

Additional file 1: **Figure S1.** *Histology of the detected MSI gallbladder carcinoma.* High magnification pictures of Hematoxylin & Eosin (HE) stained full-slide sections of the detected MSI GBC show a typical glandular, tubular, acinar, ductal morphology of pancreatobiliary subtype (A: original magnification: 200x and B: 400x). (DOCX 348 kb)

## Abbreviations

BTC: biliary tract cancer
CCA: cholangiocarcinoma
GBC: gallbladder cancer
IHC: immunohistochemistry
MMR: mismatch repair
MSI: microsatellite instability
PCR: polymerase chain reaction
