# Supplementary material for: Low frequency of mismatch repair deficiency in gallbladder cancer
Source: Diagn Pathol. 2019 May 8;14:36. doi: 10.1186/s13000-019-0813-5 (PMC6506936; doi:10.1186/s13000-019-0813-5)

**Supplemental Data to**

**Low Frequency of Mismatch Repair Deficiency in Gallbladder Cancer**

Benjamin Goeppert^1,2*^, Stephanie Roessler^1,2^, Marcus Renner^1^, Moritz Loeffler^1^, Stephan Singer^1^, Melina Rausch^1^, Thomas Albrecht^1,2^, Arianeb Mehrabi^2,3^, Monika Nadja Vogel^4^, Anita Pathil^5^, Elena Czink^2,6^, Bruno Köhler^2,6^, Christoph Springfeld^2,6^, Christian Rupp^2,6^, Karl Heinz Weiss^2,5^, Peter Schirmacher^1,2^, Magnus von Knebel Doeberitz^7^, and Matthias Kloor^7^

^1^Institute of Pathology, University Hospital Heidelberg, Im Neuenheimer Feld 224, Heidelberg, Germany

^2^Liver Cancer Center Heidelberg (LCCH)

^3^Department of General Visceral and Transplantation Surgery, University Hospital Heidelberg, Im Neuenheimer Feld 110,

Heidelberg, Germany

^4^Diagnostic and Interventional Radiology, Thoraxklinik at University Hospital of Heidelberg, Heidelberg, Germany

^5^Department of Internal Medicine IV, Gastroenterology and Hepatology, University Hospital Heidelberg, Im Neuenheimer Feld 410, Germany

^6^University Hospital Heidelberg, National Center for Tumor Diseases, Department of Medical Oncology

^7^Department of Applied Tumor Biology, Institute of Pathology, University of Heidelberg, Germany

**Supplemental Figure 1:**

*Histology of the detected MSI gallbladder carcinoma.*

High magnification pictures of Hematoxylin & Eosin (HE) stained full-slide sections of the detected MSI GBC show a typical glandular, tubular, acinar, ductal morphology of pancreatobiliary subtype (A: original magnification: 200x and B: 400x).


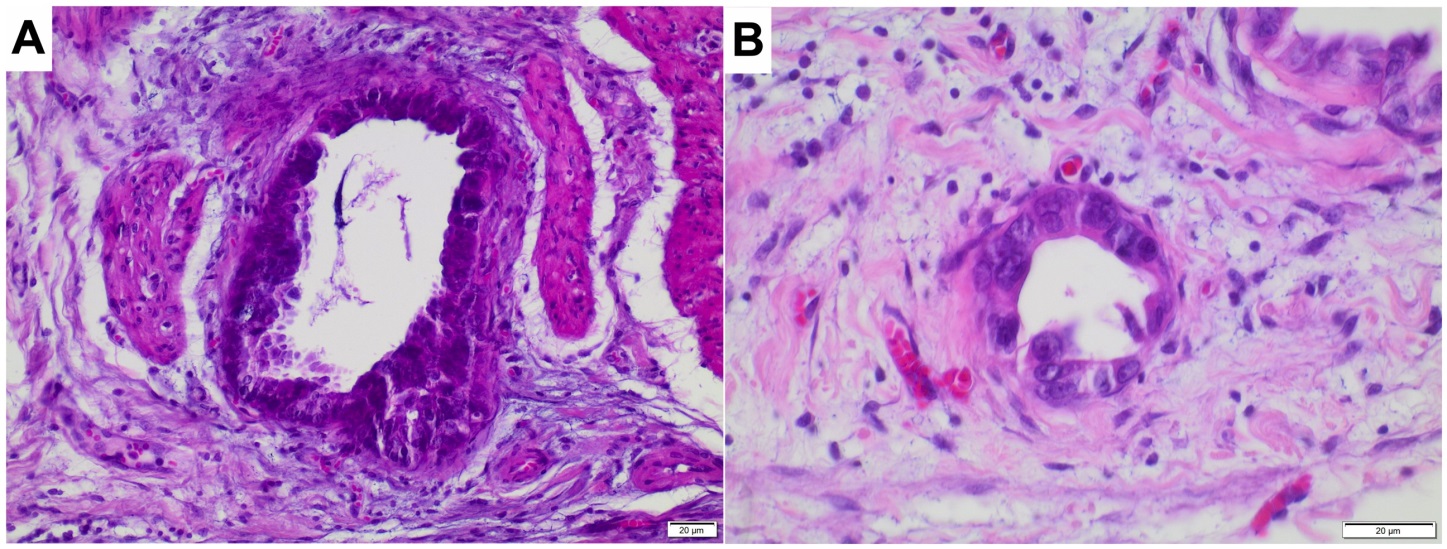

Supplement: Supplementary file 1 — Figure S1. Histology of the detected MSI gallbladder carcinoma. High magnification pictures of Hematoxylin & Eosin (HE) stained full-slide sections of the detected MSI GBC show a typical glandular, tubular, acinar, ductal morphology of pancreatobiliary subtype (A: original magnification: 200x and B: 400x). (DOCX 348 kb) [file 13000_2019_813_MOESM1_ESM.docx]
